# Inferring the molecular affinity of Indian pangolin with extant Manidae species based on mitochondrial genes: a wildlife forensic perspective

**DOI:** 10.1080/23802359.2018.1473719

**Published:** 2018-05-24

**Authors:** Ved Prakash Kumar, Ankita Rajpoot, Malay Shukla, Parag Nigam, Surendra Prakash Goyal

**Affiliations:** a Wildlife Institute of India, Dehradun, Uttarakhand, India;; b Molecular Systematics Laboratory, Zoological Survey of India, North Regional Center, Dehradun, Uttarakhand, India;; c Institute of Forensic Sciences, Gujarat Forensic Science University, Gandhinagar, Gujarat, India

**Keywords:** Pangolins, mitochondrial gene, single nucleotide polymorphic sits (SNPs), wildlife forensics, species identification and illegal trades

## Abstract

Pangolins are the world`s most trafficked mammalian species classified under family Manidae and face severe threat of extinction, largely due to the illicit trade of its parts and products, especially scales, in international markets. Pangolin scales are believed to be used in Traditional Chinese Medicines (TCM) and meat is used as delicacies in restaurants. Of the eight extant species of pangolin, morphological discrimination is easy but the situation becomes precarious once the scales and meat samples are seized and it is difficult to identify species based on morphology in such cases. However, wildlife DNA forensics has played an instrumental role in the identification of species from such type of materials.

The present study investigated that three mitochondrial genes (Cyt b, 16S rRNA, and 12S rRNA) clearly showed the variation among seven extant pangolin species (*Manis culionensis*; possibly extinct), whereas, maximum variation was obtained in cytochrome b when compared to another two mitochondrial genes.

The present study revealed that obtained SNPs based on short sequence length (Intervals) within the three mitochondrial genes will be helpful to design the short molecular marker and species-specific probe that is used in wildlife forensic for identifying pangolin species from the degraded sample. We also advocate using more than one molecular marker for species discrimination so as to minimize any false identification of the mammal's species reported in the trade. Furthermore, data generated from the study would help in strengthening the DNA database of Indian pangolin species.

## Introduction

Pangolin also known as scaly anteater is an elusive and nocturnal mammal that has the outer overlapping layer of keratinized body scales. Worldwide, eight extant species of Pangolins are recognized and divided into two groups with each of four species, i.e. Asian group (Indian pangolin; *Manis crassicaudata*, Sunda/Malayan pangolin; *M. javanica*, Chinese pangolin*; M. pentadactyla*, Philippine Pangolin; *M. culionensis,* possibly extinct*)* and African group *(*White-bellied pangolin; *Phataginus tricuspis*, Black-bellied pangolin; *P. tetradactyla*, Giant ground pangolin; *Smutsia gigantean*, and Temminck ground pangolin; *S. temminckii*), classified under sole family Manidae (Order: Pholidota) (Gaubert 2011).

Wildlife trade has emerged as one of the most lucrative markets for churning money with the least possible risk that has brought thousands of species on the brink of extinction (Smith et al. 2009). Owing to an unprecedented demand in international market, Asian pangolins are on the brink of extinction. Among them, Sunda and Chinese pangolins are listed as ‘Critically Endangered’, whereas, Indian pangolin is ‘Endangered’ under IUCN. All four African species of pangolin are listed ‘Vulnerable’ as per IUCN (IUCN 2014) (S-1). In a major decision to regulate illegal trafficking of pangolins, CITES listed all extant species are upgraded to Appendix-I from Appendix-II (CITES 2016). The Indian subcontinent has two species of Asian pangolins, i.e. Indian pangolin (widely distributed in Indian land mass) (ZSI 2002; Srinivasulu and Srinivasulu 2012) and Chinese pangolin (only present in the eastern part of India) (Mishra and Hanfee 2000). Both these species are protected under Schedule I of the Indian Wildlife (Protection) Act, 1972.

Surprisingly, during the last decade, pangolin has emerged as one of the most trafficked mammalian species in the world transcending the poaching rates compared to that of iconic species like tiger, rhino, elephant, etc. Multiple factors are responsible for the vulnerability of pangolin population viz, low reproduction rate, predation, and poaching. Pangolins are killed mostly for the want of their scales, meat, and skins to meet the international demand. Illicit trade in the markets of East Asia and South-East Asia is mainly driven by the demand for pangolin scales that are thought to be used in preparation of Traditional Chinese Medicines (TCM) and as fashion accessories/ornamentation, for spiritual and ritualistic purpose (Anon 1992; Boakye et al. 2004; Challender 2011; Mahmood et al. 2012).

However, parts and products of pangolins seized by enforcement agencies may be in the form of scales, bones, skin, or finished leather products and is difficult to assign accurate taxonomic identification to these products (Challender 2011; Du Toit et al, 2014). With an ever-escalating trans-boundary trade of pangolin, it is inevitable to have accurate species identification for addressing legal issues and better enforcement of national laws and international trades. Consequently, a DNA based approach for species identification as well as population assignments may prove to be a robust tool for wildlife law enforcement agencies (Ogden et al. 2009; Zhang et al. 2015; Rajpoot et al. 2016).

Mitochondrial markers are most validated for the identification of species, of these, cytochrome b (Cyt b), 12S ribosomal RNA (12S rRNA), 16S ribosomal RNA (16S rRNA) and cytochrome oxidase subunit I (COI) gene are routinely used for species assignment in wildlife forensics (DeSalle et al. 1993; Hsieh et al. 2001; Guha and Kashyap 2006; Alacs et al. 2009; Kumar et al. 2016a and 2018). In the present study, we described the molecular characteristics of Indian pangolin species using genes (Cyt b, 12S rRNA, and 16S rRNA) of mtDNA genome. We believe that the use of multi genes for species assignment in wildlife forensic could minimize any chance of false identification.

## Materials and Methods

### Sample collection

Fecal samples of four individuals of taxonomically identified Indian pangolin were collected during fieldwork in Rajaji Tiger Reserve, Uttarakhand, India during January 2018. Moreover, reference sequences of other extant pangolins were downloaded from NCBI Genbank (S-1).

#### Laboratory procedure

The genomic DNA was isolated from fecal samples using QIAamp DNA Stool Mini Kit (Qiagen, Germany) following manufacturer protocol. Partial fragments of the mt genes, i.e. Cyt b (350 bp) (Meyer et al. 1995), 16S rRNA (510 bp) (Mitchell et al. 1995) and 12S rRNA (402 bp) (Kocher et al. 1989) were amplified. All PCR reactions were carried out on Thermal Cycler 2720 (Applied Biosystem, USA) with a reaction set up of 15 μl volume containing 7.5 μl of 2 × Qiagen Multiplex PCR Master Mix, 0.50 μl of 10 μM of each primer pair, 1 μl of Q solution (supplied with kit), 2 μl of DNA template (∼20ng). Optimized PCR conditions and sequencing protocol as described in Kumar et al. 2014a,b were used for further processing with due positive and negative controls incorporated throughout all process. The PCR products were purified using the Exo-SAP protocol and sequenced using forward and reverse primers through outsourcing sequencing service.

#### Data analysis

Raw data generated were subjected to Sequence Analysis v5.2 (Applied Biosystem) for quality check. Data was investigated for Multiple Sequence Alignments using CLUSTAL was executed in BioEdit v.7.0.5.3 (Hall et al. 1999. The sequences procured from the four samples of Indian pangolin were cross-checked carefully with NCBI GenBank (http://blast.ncbi.nlm.nih.gov/) for species certification. For the species conclusion, we considered the rate resemblance among question and reference progression sets. Neighbour-joining tree was created using MEGA v 7 (Kumar et al. 2016b) using the neighbor-joining (NJ) methodology (Saitou and Nei 1987) with 1000 bootstrap regard (Felsenstein 1985).

## Result and Discussion

All three mt genes (Cyt b, 16S rRNA, and 12S rRNA) were successfully amplified in all four samples. NCBI Nucleotide BLAST revealed sequence identity from 99% to 100% for all four samples with Indian pangolin. Sequences generated from the present study were submitted to NCBI GenBank database (Table 1).

**Table 1.** Observed mtDNA diversity based on Cytochrome b (331bp), 16S ribosomal RNA (477bp) and 12S ribosomal RNA (383bp) genes among seven pangolin species.

Species	MC	MP	MJ	PTRI	PTET	SG	ST	Overall
Cytochrome b (331bp)								
V	16	5	6	14	26	5	11	83
C	144	140	139	147	128	128	123	
SS-SNPs	15	2	3	13	12	5	13	63
S	X	X	X	8	7	5	0	20
Genbank similarity	100%	–	–	–	–	–		
16S Ribosomal RNA (477bp)								
V	16	15	14	16	18	16	14	109
C	399	387	397	393	392	386	398	
SS-SNPs	7	11	6	8	8	7	7	53
S	X	2	X	2	1	X	2	7
Genbank similarity	99%	–	–		–	–	–	
12S Ribosomal RNA (383bp)								
V	10	6	13	15	7	12	12	75
C	317	323	31	322	317	322	321	
SS-SNPs	5	4	6	5	6	6	5	36
S	X	X	X	X	X	X	2	2
Genbank similarity	100%	–	–			–		

V: variable sites; C: conserve sites; SS-SNPs:Species-specific Single Nucleotide Polymorphic Sites; S: Singleton sites; MC: *Manis crassicaudata*; MP: *Manis pentadactyla* ; MJ: *Manis javanica*; PTRI: *Phataginus tricuspis*; PTET: *Phataginus tetradactyla*; SG: *Smutsia gigantean;* ST: *Smutsia temminckii.*

### Species variability in Cyt b gene

With reference to complete mt genome of Bos taurus (NC 008776.1), 331 bp Cyt b nucleotide sequence consisted of conserved sites (C): *n* = 226, variable sites (V):*n* = 83, singleton sites (S):*n* = 20 and fixed species-specific Single Nucleotide Polymorphic sites (SNPs): *n* = 63 (species-wise detail given in Table 1). Rather two insertion on nucleotide sites (nts) 2172-A/2208-T in Malayan pangolin and three deletions on nts 2210/2290 in Malayan pangolin; nts 2288 in Chinese pangolin, Temminck ground pangolin, and White-bellied pangolin were observed. 331bp Cyt b sequences divided into three intervals to find out the SNPs distribution in short sequences length, whereas first and second intervals divided based on 100bp nucleotide sequences and last third divided based on 180bp nucleotide sequences. The first two intervals contained *n* = 24 and *n* = 14 SNPs, while the third interval contained *n* = 25 SNPs (Table 1; S-1A; SNPs position given in Table S3-A).

**Figure 1.**
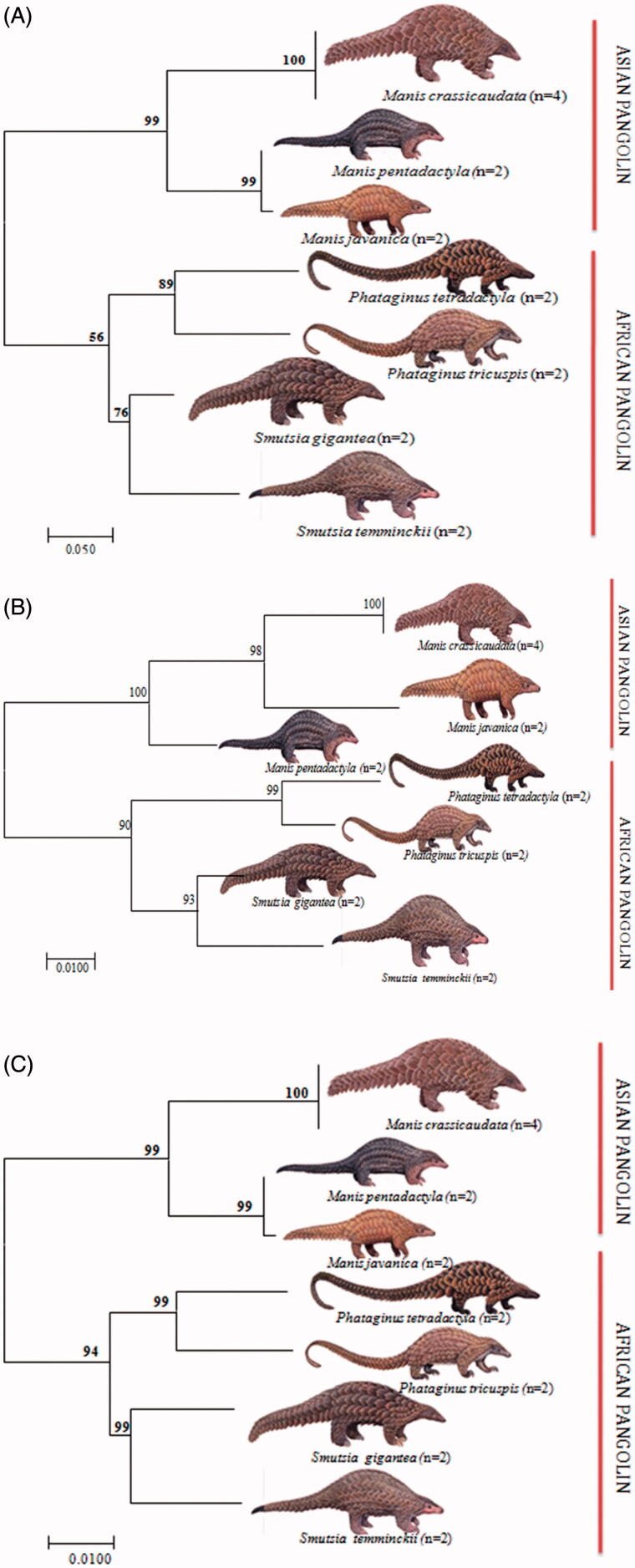
Illustrating the Cyt b (A), 16S rRNA (B) and 12S rRNA (C) sequences based Neighbour-Joining (NJ) tree analysis among seven pangolins by using MEGA 7 (Kumar et al, 2016b).

The nucleotide compositions of the sequenced region of Cyt b was as follow: T = 22.1%, C = 21.4%, A = 37.8%, and G = 18.6% among extant pangolin species. The NJ tree with 1000 bootstrap replicates showed two major clades (clade A and clade B) among seven extant pangolin species. Whereas clade A, consisted of three Asian pangolins with 99% to 100% bootstrap values, while clade B, consisted of four African pangolins with 67–90% bootstrap values (Figure 1(A)).

### Species variability in 16S rRNA gene

Similarly, in 16S rRNA gene 477 bp nucleotide sequences, consisted with C: *n* = 360, V: *n* = 109, S: *n* = 7 and species-specific SNPs: *n* = 54 (species wise details given in Table 1). Thereafter, five insertions on nts 2594-2595-TAG in Indian pangolin and Chinese pangolin; 2596-CAG in Malayan pangolin; 2605-C/A in Malayan/Blacked-bellied pangolin) and six deletions on nts 2564/2616/2728 in four African species; nts 2602 - 2604 in Chinese pangolin were observed.

Similar to Cyt b, the 477bp of 16S rRNA sequences divided into four intervals, whereas first to three divided based on 100bp nucleotide sequences, while the fourth interval divided based on 177bp nucleotide sequences. The first, second, and third intervals contained *n* = 2,*n* = 11, and *n* = 26 SNPs, while fourth intervals contained *n* = 15 SNPs. While the third has the maximum number of SNPs and it distributed among all seven extant pangolin species. Rather, no any insertion and deletion were observed (Table 1 and S1-B; SNPs position given in Table S3-A).

The nucleotide compositions of 16S rRNA are as follow: T= 25.6%, C= 20.7%, A = 33.2%, and G = 20.5% among seven pangolin species. The NJ tree topology showed similar to Cyt b whereas seven extant pangolin species grouped into two major clades (A and B). Clade A, consisted of three Asian pangolins with 98–100% bootstrap values, while in clade B, four African pangolins clustered with 67–90% bootstrap values. Interestingly, within Asian clade (A), the Indian and Malayan pangolin clustered together with 98% bootstrap values and Chinese pangolin was present as sister clades, but in Cyt b Indian pangolin preset as separate sister clade (Figure 1B).

### Species variability in 12S RNA gene

Finally, in 12S rRNA gene, 383 bp nucleotides sequences consisted with C: *n* = 289, V: *n* = 76, S: *n* = 2 and species-specific SNPs (*n* = 36) (detail given in Table 1). Despite, four insertions onnts 1065-T in all African species; nts 1157-A/G in White-bellied/Giant pangolin; nts 1170-A in White-bellied pangolin; nts 1198-A in Black-bellied pangolin, while three deletions on nts 1172/White-bellied pangolin; nts 1176/Black-bellied pangolin; nts 1195/Giant ground pangolin were observed. Similar to both previous genes, 383bp 12S rRNA sequences divided into three intervals, whereas first and second intervals divided based on 100bp nucleotide sequences, while third intervals divided based on 183bp nucleotide sequences. The first and second intervals contained *n* = 9 of each SNPs respectively, while fourth intervals contained *n* = 19 SNPs. We observed that the third intervals showed the maximum number of SNPs (*n* = 19) and distributed among seven extant pangolin species (Table 1; S1-B; SNPs position given in Table S3-A).

The nucleotide compositions of the entire sequenced region of 12S rRNA are as follow: T = 22.1%, C = 21.4%, A = 37.8%, and G = 18.6% among seven pangolin species. The tree topology (NJ) with 1000 bootstrap replicates showed consensus with that of Cyt b tree topology, where all extant pangolin species resolved with 90–100% bootstrap values, (Figure 1(C)).

## Conclusion

In the present era of conservation genetics, researchers are in pursuit of generating data with a large number of samples for species where the molecular study is poorly studied. Rather, searching for homology of questioned samples from incomplete genome would lead to false positive results. Therefore, it is advisable to generate sequences using multiple genes for the sample in question and then find on NCBI/GenBank for homologous sequences for each gene separately.

Based on NJ tree, two distinct clades were formed using all three mitochondrial genes separately, i.e. Asian clade and African clade. Analysis of genetic affinity of Indian pangolin with other extant pangolins globally revealed that Indian pangolin clustered with Malayan pangolin whereas Chinese pangolin formed a sister group with Indian pangolin using 16S rRNA. Although, similar tree topology using NJ method was observed in two mitochondrial fragments (Cyt b and 12S rRNA). This way, one can minimize the possibility of retrieving wrong sequences and identify the species of the sample in question with confidence. In view of rapidly declining pangolin population due to habitat loss and poaching as well as due to trans-boundary demand, these species need immediate attention and stringent conservation efforts for their survival.
